# Venous thromboembolism in COVID-19: A meta-summary of cases

**DOI:** 10.15537/smj.2022.43.9.20220316

**Published:** 2022-09

**Authors:** Naif Saad ALGhasab, Leen A. Altamimi, Mohammed Salem Alharbi, Sulaman S. ALMesned, Aditya K. Khetan

**Affiliations:** *From the Department of Internal Medicine (ALGhasab), Medical Collage, Ha’il University; from the Department of Medicine (Alharbi), College of Medicine, University of Ha’il, Ha’il; from the College of Medicine (Altamimi), King Saud University, Riyadh; from the Department of Surgery (ALMesned), Medical College, Qassim University, Buraydah, Kingdom of Saudi Arabia; and from the Department of Medicine (ALGhasab, Khetan), McMaster University, Canada.*

**Keywords:** COVID-19, VTE, PE, DVT, hypercoagulability, pulmonary embolism, deep venous

## Abstract

**Objectives:**

To summarize cases of venous thromboembolism (VTE), including pulmonary embolism (PE) and deep vein thrombosis (DVT) among coronavirus disease (COVID-19) patients and discuss their symptoms, diagnostic method, clinical features, and prognosis.

**Methods:**

All major databases were searched for relevant studies published between December 1, 2019 and May 5, 2021.

**Results:**

A total of 233 articles were identified, 22 describing 48 patients were included. A total of 79.1% had PE and 20.9% had DVT. Most patients were men, with a mean age of 56 years. Comorbidities were present in 70.8%, and 85.4% had at least one risk factor of VTE. 56.3% had received anticoagulation therapy. Most patients were treated in the general ward. Complications occurred in 27.1% of the patients, and recovery was achieved in 80.4%.

**Conclusion:**

Venous thromboembolism must be suspected even in patients who had received prior anticoagulant regimens or in stable cases, especially in males, the elderly, and patients with comorbidities and high D-dimer levels.

Coronavirus disease (COVID-19) is a condition induced by severe acute respiratory syndrome coronavirus 2 (SARS-CoV-2). The clinical characterization of COVID-19 varies widely, ranging from symptomless to life-threatening illnesses.^1^

Coronavirus disease is linked to a serious illness course in approximately 23% of infected people and death in approximately 6% of those affected. Individuals with comorbidities and clinical symptoms related to COVID-19 severity should be regularly evaluated, and high-risk groups should be the focus of preventative efforts.^2^ Inflammation and coagulation malfunction were found to be risk factors for a complicated disease course.^3^ Pulmonary embolism (PE) and deep veinous thrombosis (DVT) are examples of venous thromboembolism (VTE), which has been addressed frequently as a common cardiovascular complication among patients with COVID-19. However, higher levels of thrombotic markers have been discovered to be linked to poor clinical outcomes.^4^

A recently published meta-analysis^5^ revealed that the combined incidence rates of VTE, PE, and DVT in hospitalized COVID-19 patients were 21%, 15%, and 27%, accordingly. These rates were higher among patients in the intensive care unit (ICU), and the findings suggest that anticoagulant use was not related to a reduced risk of mortality. Thus, evaluation of the prophylactic and therapeutic anticoagulation roles of COVID-19 is required.^5^ Another meta-analysis of 1,988 patients revealed a significant prevalence rate of thromboembolic disorders among COVID-19 patients. In these patients, the prevalence rates of VTE was 31.3%, DVT was 19.8%, and PE was 18.9%. This highlights the importance of appropriate screening methods and antithrombotic implantation to avoid fatal and severe consequences.^6^

The intrinsic pathophysiology of COVID-19-associated hypercoagulability and coagulation disorders is not fully understood. However, several hypotheses have been proposed to explain possible pathophysiological mechanisms, including receptor binding, complement activation, cytokine storm, and direct viral endothelial damage.^7,8^

The purpose of this meta-case summary is to review and summarize published cases of VTE, either PE or DVT, in patients with COVID-19 and to discuss their symptoms, diagnostic method, clinical features, and prognosis. Also, to shed light on different management practices for VTE.

## Methods

We carried out a search on major data bases such as Web of Science, PubMed, Medline, and Scopus to identify all relevant articles published between December 1, 2019, and May 5, 2021. The search algorithms were “COVID” or “coronavirus” or ‘SARS-CoV-2” and “venous thromboembolism (VTE)” or “pulmonary embolism (PE)” or “deep vein thrombosis (DVT)”. The search was not limited by publication date or language.

Clinical studies with the following criteria were considered for inclusion were patient with confirmed diagnosis of COVID-19 and VTE has been detected. There were no limits on the type of publication, both case reports and case series were included. Studies that did not report cases of VTE, DVT, or PE, and animal studies were excluded. Two researchers independently screened titles and abstracts, and any inconsistencies were resolved by a discussion. The data was obtained into a standard format, that included: patient features, duration and severity of coronavirus infection, type, and symptoms of VTE, investigations, management, and prognosis. The studies were classified according to the type of VTE treatment and the severity of the coronavirus infection. The qualities of the studies were assessed using the Newcastle-Ottawa scale, a validated measure for case reports and case series.

### Statistical analysis

Findings are summarized in tables and in a meta-summary. Flowcharts of the included studies are presented in diagrams and in a summary of pooled VTE cases. In the meta-summary, patients with verified VTE cases were presented in compared to patients with potential instances of the disease. Frequency and percentage are used to present categorical variables. Mean, standard deviation (SD), median and interquartile range were used to represent the continuous variables. Statistical analysis carried out using Statistical Package for Social Sciences, version 25.0. (IBM Corp. Armonk, NY).

## Results

**Figure 1:**
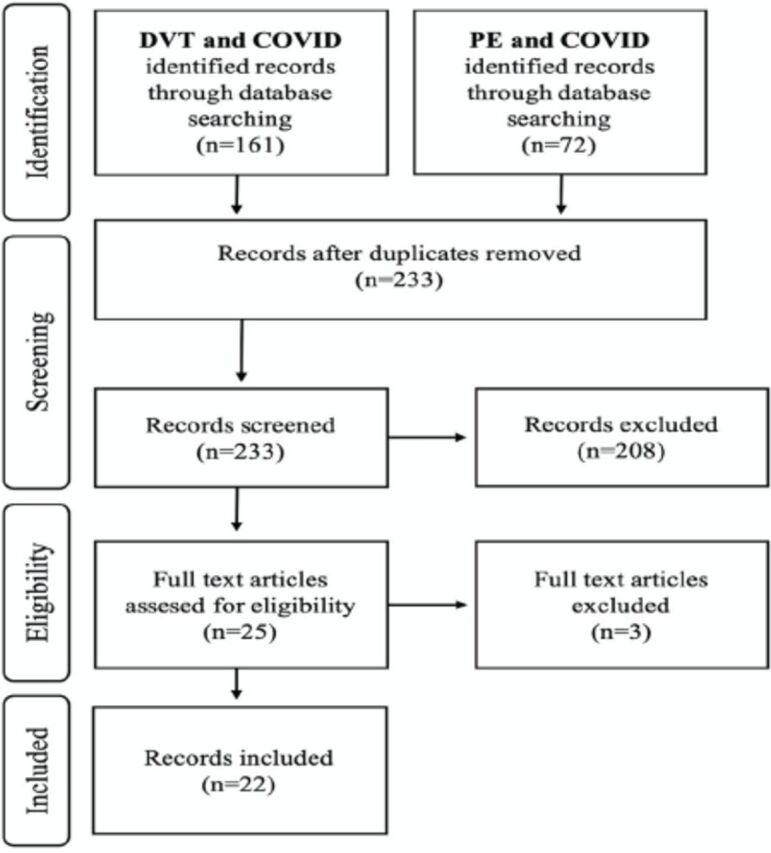
- Flow chart of study selection.

During the search, 233 articles were identified. The 208 articles were eliminated according to their titles and abstracts. Three full-text articles were eliminated due to a lack of VTE description in the remaining 25 publications. Thus, 22 studies^9-30^ representing 48 cases were included in the meta-summary. Fourteen studies were carried out in the United States,^9,10,15-19,21-24,28-30^ 7 in Europe,^11-14,20,25,26^ and one was carried out in the United Arab Emirates.^27^ Of the studies, 12 were case reports and 10 were case series. Figure 1 illustrates an overview of the article selection process.

The features of the published cases of COVID-19-associated VTE are presented in Table 1. The patients varied in age from 20 to 92, with a mean of 56 years. Males accounted for 70.8% of the total number of patients. The following were the most prevalent comorbidities: hypertension (39.6%), diabetes mellitus (27.1%), and dyslipidemia (22.9%). Most patients presented with risk factors of VTE, such as advanced age (79.2%), overweight/obesity (12.5%), and prior VTE events (6.2%).

**Table 1:** - Characteristics of published cases of coronavirus associated venous thromboembolism (N=48).

Variable	Valid= (n/48)	n (%)	Confirmed PE (n=38)	Confirmed DVT (n=10)	*P*-value
Age (years)**	48	56±17.17	56±16.90	58.00±19.07	0.796
Male	48	34 (70.8)	26 (68.4)	8 (80.0)	0.745
***Presenting symptoms of COVID-19***	45				
Fever		29 (60.4)	24 (63.2)	5 (50.0)	0.694
Chills		3 (6.2)	2 (5.3)	1 (10.0)	1.000
Cough		29 (60.4)	26 (68.4)	3 (30.0)	0.065
SOB, dyspnoea		24 (50.0)	22 (57.9)	2 (20.0)	0.076
Fatigue, malaise		9 (18.8)	7 (18.4)	2 (20.0)	1.000
Myalgia		9 (18.8)	6 (15.8)	3 (30.0)	0.569
Headache		6 (12.5)	5 (13.2)	1 (10.0)	1.000
New loss of taste or smell (anosmia)		1 (2.1)	1 (2.6)	0 (0.0)	1.000
Nausea or vomiting		2 (4.2)	2 (5.3)	0 (0.0)	1.000
Diarrhea		6 (12.5)	5 (13.2)	1 (10.0)	1.000
Chest pain (pleuritic pain)		5 (10.4)	4 (10.5)	1 (10.0)	1.000
***Presenting symptoms/signs of PE***	38				
Chest pain		9 (18.8)	8 (21.1)	1 (10.0)	0.733
Tachycardia		2 (4.2)	1 (2.6)	1 (10.0)	0.882
Shortness of breath (dyspnea)		30 (62.5)	27 (71.1)	3 (30.0)	0.044
Hypoxia (O2 saturation <90%)		9 (18.8)	9 (23.7)	0 (0.0)	0.211
***Presenting symptoms of DVT***	9				
Unilateral erythematous swelling of the lower extremity		4 (8.3)	1 (2.6)	3 (30.0)	0.032
Leg pain or tenderness of the thigh or calf		3 (6.2)	0 (0.0)	3 (30.0)	0.006
***Comorbidities***	34				
Diabetes mellitus		13 (27.1)	10 (26.3)	3 (30.0)	1.000
Hypertension		19 (39.6)	14 (36.8)	5 (50.0)	0.694
Dyslipidemia		11 (22.9)	8 (21.1)	3 (30.0)	0.86
Asthma		6 (12.5)	6 (15.8)	0 (0.0)	0.42
Atrial fibrillation		5 (10.4)	3 (7.9)	2 (20.0)	0.594
Coronary artery disease		4 (8.3)	1 (2.6)	3 (30.0)	0.032
Congestive heart failure		4 (8.3)	2 (5.3)	2 (20.0)	0.391
Hypothyroidism		4 (8.3)	2 (5.3)	2 (20.0)	0.391
Obstructive sleep apnea		2 (4.2)	2 (5.3)	0 (0.0)	1.000
***Risk factors for VTE***	41				
Overweight/ obese		6 (12.5)	6 (15.8)	0 (0.0)	0.42
Immobility		1 (2.1)	1 (2.6)	0 (0.0)	1.000
Prior VTE		3 (6.2)	1 (2.6)	2 (20.0)	0.199
Advanced age (>40 years)		38 (79.2)	30 (78.9)	8 (80.0)	1.000
Hospitalization, surgery		2 (4.2)	1 (2.6)	1 (10.0)	0.882
Family history of VTE, stroke		2 (4.2)	1 (2.6)	1 (10.0)	0.882
Recent or recurrent cancer		1 (2.1)	0 (0.0)	1 (10.0)	0.468
***Serology***	48				
D-dimer >500ng/ml		38 (79.2)	30 (78.9)	9 (90.0)	0.403
Lactate (mml/L) **		4.93±4.40	5.74±4.39	0.90	N/A
LDH (units/L) **		454.89 ±222.34	454.89±222.34	0 (0.0)	N/A
Ferritin >1000 ng/ml		8 (16.7)	8 (21.1)	0 (0)	N/A
***EKG***	4	4 (8.3)	4 (10.5)	0 (0.0)	0.668
Normal sinus rhythm		3 (6.3)	3 (7.9)	0 (0.0)	
Sinus tachycardia		1 (2.1)	1 (2.6)	0 (0.0)	
***Echocardiogram***	11				
Right ventricular dysfunction		6 (12.5)	6 (15.8)	0 (0.0)	0.42

Values are presented as number and percentages (%). **Median, N/A- not available, COVID-19: coronavirus disease 2019, PE: pulmonary embolism, DVT: deep vein thrombosis, VTE: venous thromboembolism, EKG: electrocardiogram, ICU: intensive care unit, LDH: lactic acid dehydrogenase, O2: Oxygen, SOB: shortness of breath

**Table 1:** - Characteristics of published cases of coronavirus associated venous thromboembolism (continuation).

Variable	Valid= (n/48)	Total cases (n=48)	Confirmed PE (n=38)	Confirmed DVT (n=10)	*P*-value
***Covid management***	25				
Hydroxychloroquine		15 (31.2)	15 (39.5)	0 (0.0)	0.044
Azithromycin		6 (12.5)	6 (15.8)	0 (0.0)	0.42
Amoxicillin clavulanate		4 (8.3)	4 (10.5)	0 (0.0)	0.668
Dexamethasone		5 (10.4)	3 (7.9)	2 (20.0)	0.594
Cefepime		5 (10.4)	3 (7.9)	2 (20.0)	0.594
Linezolid		3 (6.2)	2 (5.3)	1 (10.0)	1.000
***Outcome***	48				
Recovery or discharge		37 (80.4)	30 (81.1)	7 (77.8)	1.000
Death		9 (19.6)	7 (18.9)	2 (22.2)	1.000
Undetermined		2 (4.1)	1 (2.6)	1 (0.10)	0.882
***Severity of Covid infection***	28				
ICU admissions		10 (20.8)	9 (23.7)	1 (10.0)	0.663
Average length of ICU admission (days)		3.31 (±8.25)	1.84 (±3.54)	40.00 (1 patient)	N/A
Average length of hospital admission		4.90 (±4.45)	4.83 (±4.67)	5.50 (±2.12)	0.847
Complication during admission		13 (27.1)	10 (26.3)	3 (30.0)	1.000
Time of dx till VTE discovery**		10.30 (±7.92)	10.82 (±8.28)	8.11 (±6.11)	0.363
Duration of COVID symptoms**	43	7.91 (±7.03)	7.97 (±7.45)	7.57 (±4.65)	0.892

Values are presented as number and percentages (%). **Median, N/A- not available, COVID-19: coronavirus disease 2019, PE: pulmonary embolism, DVT: deep vein thrombosis, VTE: venous thromboembolism, dx: diagnosis, ICU: intensive care unit

Coronavirus disease-19 was most associated with the following symptoms: fever (60.4%), cough (60.4%), shortness of breath (50%), fatigue (18.8%), myalgia (18.8%), headache (12.5%,), diarrhea (12.5%), and chest pain (10.4%). Other reported symptoms included chills, nose congestion, nausea or vomiting, sore throat, and loss of taste or smell.

Of the 48 patients, 79.1% had confirmed cases of PE, and 68.4% were males. The most common presenting symptoms of PE were dyspnea (62.5%) and chest pain (18.8%), whereas the most common signs were hypoxia (18.8%) and tachycardia (4.2%). The remaining 10 patients (20.9%) were confirmed to have DVT, most of whom were men (80%). The most common presenting symptoms of DVT were unilateral swelling of the lower extremity (8.3%) and leg pain or tenderness (6.2%). Only 3 patients developed PE after DVT diagnosis.

Of the 48 patients with confirmed COVID-19 infection, 15 were treated with hydroxychloroquine; 6, azithromycin; 5, steroids (dexamethasone); 5, cefepime; 5, convulsant plasma; 4, amoxicillin clavulanate; 3, linezolid; and 2, oseltamivir.

Most patients were treated in the general ward, with a mean length of hospital admission of 4.90±4.45 days. Only 20.8% of the patients were transferred to the ICU. The average duration of ICU admission was 3.3±8.25 days. Recovery or hospital discharge was achieved in 80.4% of patients with COVID-19, whereas 19.6% died.

The investigation results and imaging characteristics of the confirmed cases of COVID-19-associated VTE are presented in Table 2. D-dimer levels were found to be increased in 78.9% of patients with PE and 90% of those with DVT. Troponin levels (troponins I or T) were found to be high in 13% of the patients. The N-terminal pro-B-type natriuretic peptide (NT-pro-BNP) level was underreported and raised in 7% of the patients. In most patients, other inflammatory indicators such as white cell count, ferritin, and C-reactive protein levels were raised.

**Table 2:** - Imaging characteristics of confirmed cases of coronavirus associated venous thromboembolism.

Author name	Age/Gender	D-dimer >500 ng/mL**	Chest x-ray with pneumonia		Echocardiogram	CTPA	Duplex ultrasound / CUS	Other	
Davis, Kenyani	34 M	N/A	+		N/A	Right Lower Lobe PE	N/A	N/A	
Aoi et al	70 F	+	N/A		Dilated RV and clot in transit	Saddle PE	N/A	N/A	
Brüggemann et al	57 M	++	+		N/A	Right pulmonary artery and bilateral sub-segmental PE	N/A	CT Brain showed right frontal lobe infarction	
Colombo et al	73 F	N/A	+		N/A	Bilateral PE	Normal	N/A	
Delcros et al	31 M	++	N/A		N/A	Bilateral PE	N/A	CT venography showed a femoropopliteal DVT expanding to the subrenal vena cava	
Fiorini et al	26 F	+	N/A		Normal	Bilateral sub-segmental PE	Normal	N/A	
Haider et al	46 F	+	+		N/A	Bilateral PE (segmental and sub-segmental)	N/A	N/A	
Kasinathan et al	20 F	+	+		N/A	Bilateral PE	N/A	N/A	
Mene-Afejuku et al	67 M	++	+		N/A	Bilateral PE	N/A	N/A	
	58 F	++	+		N/A	Large saddle PE extending to lobar, segmental, and subsegmental pulmonary arteries.	N/A	N/A	
	89 F	++	N/A		N/A	Bilateral PE	N/A	N/A	
	82 F	++	+		N/A	Bilateral PE	N/A	N/A	
Akel et al	28 F	++	N/A		Dilated RV with interventricular septal flattening	Bilateral extensive PE	N/A	N/A	
	52 M	++	+		N/A	Bilateral PE	N/A	N/A	
	62 M	++	N/A		McConnell’s sign	Bilateral PE	N/A	N/A	
	49 M	++	N/A		RV dilatation along with systolic and diastolic flattening of the septum	Right segmental PE	N/A	N/A	
	59 F	+	+		N/A	Bilateral PE	N/A	N/A	
	69 M	++	N/A		N/A	Large bilateral PE	N/A	N/A	
Fortuzi et al	52 M	++	+		N/A	Right PE	N/A	N/A	
	74 F	++	N/A		N/A	Bilateral PE	N/A	N/A	
	31 M	+	N/A		N/A	Right sub-segmental PE	N/A	N/A	
Kanso et la	68 M	++	+		N/A	Right segmental PE	N/A	N/A	
	62 M	++	+		N/A	Left segmental PE	N/A	N/A	
Lewis et al	77 M	++	N/A		N/A	Bilateral PE	N/A	N/A	
	70 M	+	N/A		N/A	N/A	Partial occlusion in popliteal and femoral veins	N/A	
	76 M	++	N/A		N/A	Right segmental and sub-segmental PE	N/A	N/A	
	80 M	N/A	N/A		N/A	N/A	DVT of femoral vein	N/A	
	92 M	+				Care withdrawn			
Manek et al	66 M	++	N/A	N/A		Bilateral PE	DVT of the left femoral vein	N/A	
Mangala et al	55 M	+	N/A	N/A		Right segmental PE.	N/A	EKG showed normal sinus rhythm
	67 F	N/A	+	N/A		PE in right upper lobe pulmonary artery and segmental branches of right lower lobe pulmonary artery	N/A	EKG showed normal sinus rhythm
Nelson, et al	61 M	+	+	N/A		Right segmental PE	Multiple areas of turbulent flow in lower extremity	N/A
	54 M	++	+	N/A		Negative for PE	Turbulent blood flow in the right lower extremity and right calf vein thrombosis	N/A
Overstada et al	55 M	++	N/A	N/A		N/A	DVT in the left leg	N/A
	39 M	+	N/A	N/A		Bilateral PE	N/A	N/A
	57 M	+	N/A	N/A		Left segmental PE	N/A	N/A
	55 M	++	+	N/A		Bilateral PE	N/A	N/A
Sakr et al	66 M	++	+	Dilated RV and paradoxical septal motion		Bilateral PE	Right femoral vein thrombosis	N/A
	65 M	N/A	+	Mild dilatation of the RV with preserved LV function		Right segmental PE	Normal	N/A
	56 M	N/A	+	N/A		Right segmental PE	Normal	N/A
	41 M	N/A	+	Acute right heart failure with paradoxical septal motion and large thrombus in the right pulmonary artery		N/A	Thrombosis of the left femoral vein	N/A
	49 M	N/A	+	N/A		Right segmental PE	Normal	N/A
Salam et al	36 M	+	+	McConnell’s sign with septal flattening		Saddle PE with significant clot burden.	Normal	EKG showed sinus tachycardia
Sethi et al	44 M	++	+	EF of 45%, severely dilated and reduced RV systolic function with a flattening of the septum.		N/A	Normal	N/A
Singh et al	69 F	+	N/A	N/A		N/A	N/A	CTA showed thrombotic occlusion in tibial arteries on the right leg, aortic thrombus in the aorta, with evidence of splenic infarct.
	33 M	+	N/A	N/A		N/A	N/A	CTA showed occlusive thrombus at the aortic bifurcation with near-complete occlusion of right common iliac arteryy.
	69 F	+	N/A	EF of 25%-35% and evidence of a large LV thrombus at the apex.		N/A	N/A	N/A
Uppuluri et al	32 M	N/A	+	N/A		Left segmental and subsegmental PE.	N/A	EKG showed normal sinus rhythm

N/A: not available, RV: right ventricle, EF: ejection fraction, LV: left ventricle, PE: pulmonary embolism, Echo: echocardiogram, CT: computed tomography, CTPA: CT pulmonary angiography, CUS: compression ultrasonography, CTA: computed tomography angiography, EKG: electrocardiogram, F: female, M: male, **(+) means >500 ng/mL, (++) means >5000 ng/mL.

Chest radiography was performed for most of the patients, of whom 52.1% had signs of pneumonia, such as consolidation, ground-glass opacities, and infiltration. Echocardiography results were reported in only 11 patients, 10 of whom had abnormalities consistent with moderate to severe pulmonary embolism. Features included dilated right ventricle, systolic septal flattening, hypercontractile apex, and right ventricle dysfunction. An electrocardiography result was reported in only 4 patients, most of whom had a normal sinus rhythm.

The diagnosis of PE was confirmed using computed tomography (CT) and pulmonary angiography (CTPA), while the diagnosis of DVT was confirmed with duplex ultrasonography, compression ultrasonography (CUS), or CT venography in most cases. The most common CTPA finding was bilateral PE, followed by right- and then left-sided pulmonary embolism.

The types of anticoagulants used to treat the confirmed cases of COVID-19-associated VTE are presented in Table 3. Of the 48 patients, 56.3% had received anticoagulant therapy prior to VTE diagnosis (either for the treatment of a preexisting medical condition or as a prophylaxis during hospitalization). Of the patients, 51.9% were taking low-molecular-weight heparins (LMWHs) such as nadroparin and enoxaparin, 25.9% were taking heparin, and 22.2% were taking novel oral anticoagulants (NOACs) such as apixaban and rivaroxaban. During hospital admission, after VTE diagnosis, the most used medications were LMWHs (62.5%), intravenous heparin (22.9%), tissue plasminogen activators (14.6%), NOACs (10.4%), and venoarterial extracorporeal membrane oxygenation (4.2%). Upon discharge, NOACs and LMWHs were prescribed to 58.3% and 18.8% of the patients, respectively. The other medications prescribed included warfarin and acetylsalicylic acid. The discharge plans of the remaining patients (22.9%) were unclear owing to either death or car withdrawal, or because they were not fully reported.

**Table 3:** - Anti-coagulation type used to treat confirmed cases of coronavirus associated venous thromboembolism.

Author name	Age/Gender	Type of anticoagulant			Outcome	Complications
		Prior to VTE	VTE treatment (during hospitalization)	Upon discharge		
Kenyani Davis	34 M	N/A	N/A	NOAC	Recovery or discharge	N/A
Aoi et al	70 F	Heparin	IV Heparin	N/A	Death	Cardiac arrest
Brüggemann et al	57 M	LMWH (nadroparin)	LMWH (tinzaparin)	N/A	Recovery or discharge	Ischemic stroke
Colombo et al	73 F	LMWH (enoxaparin)	LMWH (enoxaparin)	NOAC	Recovery or discharge	Obstructive shock
Delcros et al	31 M	LMWH (enoxaparin)	LMWH (enoxaparin)	LMWH (enoxaparin)	Recovery or discharge	N/A
Fiorini et al	26 F	N/A	LMWH (enoxaparin)	NOAC	Recovery or discharge	N/A
Haider et al	46 F	Heparin	LMWH (enoxaparin)	NOAC	Recovery or discharge	N/A
Kasinathan et al	20 F	N/A	LMWH (enoxaparin)	LMWH (enoxaparin)	Recovery or discharge	N/A
Mene-Afejuku et al	67 M	Heparin	NOAC	NOAC	Recovery or discharge	N/A
	58 F	Heparin	LMWH (enoxaparin)	NOAC	Recovery or discharge	N/A
	89 F	Heparin	IV Heparin	NOAC	Recovery or discharge	N/A
	82 F	LMWH (enoxaparin)	LMWH (enoxaparin)	NOAC	Recovery or discharge	N/A
Akel et al	28 F	LMWH (enoxaparin)	tPA, LMWH (enoxaparin)	NOAC	Recovery or discharge	N/A
	52 M	LMWH (enoxaparin)	LMWH (enoxaparin)	NOAC	Recovery or discharge	N/A
	62 M	N/A	tPA, IV heparin	NOAC	Recovery or discharge	N/A
	49 M	LMWH (enoxaparin)	LMWH (enoxaparin)	NOAC	Recovery or discharge	N/A
	59 F	LMWH (enoxaparin)	LMWH (enoxaparin)	NOAC	Recovery or discharge	N/A
	69 M	Heparin	IV Heparin	NOAC	Recovery or discharge	N/A
Fortuzi et al	52 M	Heparin	IV Heparin	N/A	Death	AKI, Septic shock
	74 F	LMWH (enoxaparin)	LMWH (enoxaparin)	NOAC	Recovery or discharge	N/A
	31 M	N/A	NOAC	NOAC	Recovery or discharge	N/A
Kanso et la	68 M	LMWH (enoxaparin)	LMWH (enoxaparin)	Warfarin	Recovery or discharge	N/A
	62 M	LMWH (enoxaparin)	LMWH (enoxaparin)	NOAC	Recovery or discharge	N/A
Lewis et al	77 M	NOAC (Apixaban)	LMWH (enoxaparin)	N/A	Death	Multi-system organ failure
	70 M	NOAC (Apixaban)	LMWH (enoxaparin)	NOAC (Apixaban), LMWH (enoxaparin), loaded warfarin	Recovery or discharge	N/A
	76 M	NOAC (Rivaroxaban)	LMWH (enoxaparin)	NOAC (Rivaroxaban)	Recovery or discharge	N/A
	80 M	NOAC (Rivaroxaban)	LMWH (enoxaparin)	LMWH (enoxaparin)	Death	N/A
	92 M	NOAC (Apixaban)	Care withdrawn	Care withdrawn	Death	Left-sided facial droop, left-sided hemiplegia and stroke
Manek et al	66 M	N/A	IV heparin	NOAC (Apixaban)	Recovery or discharge	N/A
Mangala et al	55 M	LMWH (enoxaparin)	LMWH (enoxaparin)	NOAC (Apixaban)	Recovery or discharge	aspiration pneumonia
	67 F	LMWH (enoxaparin)	IV heparin	NOAC (Apixaban)	Recovery or discharge	N//A
Nelson et al	61 M	N/A	IV heparin	N/A	Death	Septic shock
	54 M	N/A	tPA, LMWH (enoxaparin)	N/A	Death	N/A.
Overstada et al	55 M	N/A	NOAC (Apixaban)	N/A	Recovery or discharge	N/A
	39 M	N/A	NOAC (Apixaban)	NOAC (Apixaban)	Recovery or discharge	N/A
	57 M	N/A	NOAC (Apixaban)	NOAC (Apixaban)	Recovery or discharge	N/A
	55 M	N/A	LMWH (Dalteparin)	LMWH (Dalteparin)	Recovery or discharge	N/A
Sakr et al	66 M	N/A	tPA and IV heparin	N/A	Death	Progressive multi-organ failure
	65 M	N/A	LMWH (enoxaparin)	LMWH	Recovery or discharge	N/A
	56 M	N/A	LMWH (enoxaparin)	LMWH	Recovery or discharge	N/A
	41 M	N/A	tPA, VA -ECMO, LMWH (tinzaparine)	LMWH	Recovery or discharge	Ventilator-associated pneumonia, severe hypoxemia.
	49 M	LMWH (enoxaparin)	LMWH	N/A	Death	Necrotizing pneumonia
Salam et al	36 M	N/A	tPA , LMWH (enoxaparin)	NOAC (Apixaban)	Recovery or discharge	N/A
Sethi et al	44 M	N/A	tPA, LMWH (enoxaparin), VA -ECMO	LMWH	Recovery or discharge	Bleeding within the oropharynx
Singh et al	69 F	N/A	IV heparin	NOAC (Rivaroxaban) with low dose ASA	Recovery or discharge	N/A
	33 M	N/A	IV heparin	NOAC (Rivaroxaban)	Recovery or discharge	N/A
	69 F	NOAC (Apixaban)	LMWH	N/A	Undetermined	Massive stroke in the territory of the left middle cerebral artery and clinically deteriorated.
Uppuluri et al	32 M	N/A	LMWH (enoxaparin)	NOAC (Apixaban)	Undetermined	N/A

VTE: venous thromboembolism, N/A: not available, M: male, F: female, LMWH: low molecular weight heparin, IV: intravenous; NOAC: novel oral anticoagulants, VA-ECMO: venoarterial extracorporeal membrane oxygenation, AKI: acute kidney injury, tPA: tissue plasminogen activator, ASA: acetylsalicylic acid (aspirin)

Complications such as stroke, respiratory failure, shock, acute kidney injury, ventilator-associated pneumonia, bleeding in the oropharynx, and multiorgan dysfunction were found in 27.1% of the patients during admission and were associated with older age and poor prognosis.

## Discussion

This meta-analysis, which included 48 patients, revealed that VTE occurred in a significant number of patients with COVID-19, regardless of whether they were treated in the general ward or admitted to the ICU. Pulmonary embolism episodes were more common than DVT episodes. Our results support the potentially higher incidence rates of COVID-19-associated VTE in males, the elderly, and those with hypertension, diabetes mellitus, dyslipidemia, and elevated D-dimer levels. However, despite some complications during admission, most patients had a good prognosis and were discharged alive. This study also discussed diagnostic procedures and management plans for patients with COVID-19, including antiviral medicines and various anticoagulant regimens.

Pulmonary embolism is usually triggered by DVT, alhtough the presence of PE does not always indicate the existence of DVT. Thus, the detection of both acute PE and DVT in patients with COVID-19 is essential. The diagnosed of PE in COVID-19-affected patients may have a concomitant lower-extremity DVT occurrence in less than 50% of cases. Even though lower limb DVT is the predominant type, a recent retrospective review of 257 moderate-to-severe COVID-19 patients found that upper extremity deep vein thrombosis (UEDVT) occurred in approximately 10.9% of the patients (9% CI, 7.1-14.7) especially when other risk factors like venous catheters or venous stasis existed. Furthermore, when compared to individuals who did not require ventilation, using continuous positive airway pressure (CPAP) raised the incidence of UEDVT by 6 times. The presence of UEDVT has been linked to a poor prognosis and death.^31^

In patients with COVID-19, abnormal coagulation results such as disseminated intravascular coagulation and markedly elevated levels of D‐dimer and fibrin degradation products are linked with poor outcomes and death.^32^ A published systematic review assessed the diagnostic precision of D-dimer tests for PE and found that D-dimer levels >500 and >1000 µg/L showed high sensitivity (96% and 91%, respectively) but low specificity (10% and 24%, respectively) in the diagnosis of PE in patients with COVID-19, which is equivalent with the sensitivity of D-dimer levels in patients without COVID-19.^33^

On the other hand, a multicenter retrospective analysis that included 451 hospitalized patients with COVID-19 who underwent an ultrasonographic evaluation revealed that 14% of the patients had acute DVT. The D-dimer level was the most important influencing factor of the risk of DVT, and D-dimer levels > 5000 ng/mL were considered beneficial for predicting DVT. Therefore, in the early stages of COVID-19, regular D-dimer monitoring may be beneficial.^34^ In our summary, most patients with PE and DVT had a positive D-dimer test result (>500 µg/L; Table 1). However, in consideration of the high range of occurrence of VTE in patients with COVID-19, a high standard of doubt is essential, regardless of the D-dimer value.

A recent systematic review was conducted to identify the lung histopathological features of COVID-19 and to see how they relate to another previous viral pandemic. The review revealed that the presence of microthrombi in capillaries, small and medium vessels was a frequent histopathological pattern in lung pathologies in patients with COVID-19 (57%) and SARS (58%) in comparison to those with H1N1 influenza (24%). This points to a link between coronaviruses and microthrombi.^35^

In a recent retrospective study in Italy, 476 hospitalized COVID-19 patients from the first (n=316) and second (n=160) COVID-19 waves were compared. There were no significant variations in baseline features, admission biomarkers, severity metrics, or lethality between the first and second COVID-19 waves of patients admitted to a particular hospital and undergoing standard-dose thromboprophylaxis. However, a larger percentage of patients in the second wave had acute respiratory distress syndrome (46.3%) compared to the first wave (29.1%). The median length of hospitalization was longer in the second wave (17 days) compared to the first wave (10 days). In addition, the second wave had a greater rate of VTE. Possible explanations include a longer stay in the hospital, more diagnostic testing, and increased knowledge of thromboembolic problems. This emphasizes the fact that VTE identification is not completely reliant on the presence or absence of common signs and symptoms; rather, more frequent imaging is linked to more VTE incidents diagnoses.^36^

Several studies have indicated the risk of VTE since the COVID-19 epidemic started in both inpatient and outpatient patients with COVID-19. According to the recommendations of the VAS European Independent Foundation in Angiology/Vascular Medicine, patients who undergo home therapy for COVID-19 should begin thromboprophylaxis with LMWH, rivaroxaban, or betrixaban. For hospitalized patients, thromboprophylaxis should be administered with weight-adjusted intermediate doses of LMWH. For the treatment of VTE or hypercoagulability during hospital admission, LMWHs are preferable to unfractionated heparins or NOACs. After discharge, prolonged thromboprophylaxis with LMWH, rivaroxaban, or betrixaban is recommended.^37^ National Institute for Health and Care Excellence (NICE) recommendations which were recently released, suggested administering LMWH at a therapeutic dosage for people with COVID-19 who need oxygenation without an increased risk of bleeding.^38^ We found that LMWHs (enoxaparin, dalteparin, and nadroparin) were the most frequently used medications for prophylaxis and the treatment of hospitalized patients, whereas NOACs (apixaban and rivaroxaban) were the most frequently used medication after discharge.

In a recently published prospective cohort study with almost 6195 patients with COVID-19, 598 patients were hospitalized upon diagnosis, and 5597 were managed as outpatients. Of the outpatients, 2.9%received outpatient anticoagulation (OPAC). The likelihood of being admitted to the hospital was lowered by 43% in the patients who were receiving OPAC before the diagnosis of COVID-19. Unfortunately, owing to the small number of users, the precision of the effect of the type and dosage of the anticoagulants used, such as warfarin, NOACs, and enoxaparin, were inadequate. Furthermore, in hospitalized COVID-19 patients, failure to start anticoagulation or to continue OPAC was linked to a greater mortality risk.^39^ In 89.6% of the cases in our summary, the COVID-19 signs and symptoms lasted for 7.91±7.03 days, and VTE was discovered 10.30±7.92 days after diagnosis. This emphasizes the significance of initiating thromboprophylaxis upon diagnosis.

Owing to the ongoing pandemic, it is essential to promote awareness of VTE as a possible complication of COVID-19 infection. Many approaches have been used to reduce the likelihood of VTE occurrence and to control confirmed cases. However, failure of thromboprophylaxis and the presence of VTE in patients with COVID-19 admitted to ICUs imply that rather than a broad treatment plan, a customized approach tailored to the severity and stage of the disease should be used.^40^ In France, a comprehensive multi-center retrospective study was carried out to investigate the impact of intermediate-dose compared to standard prophylactic anticoagulation (AC) in COVID-19 patients admitted to medical wards. In-hospital mortality was not considerably different.^41^ In our meta-case summary, 56.3% of the patients had received anticoagulant therapy prior to hospitalization. However, it did not reduce the incidence rate of VTE, or complications found upon admission, which suggests a prophylaxis failure.

Novel oral anticoagulants are among the commonly used anticoagulants and have demonstrated acceptable efficacy/safety features; nonetheless, some studies have shown drug-drug interactions between NOACs, and specific antiviral drugs used in patients with COVID-19.^38^ A recent study compared 13,003 patients with COVID-19 who received NOAC therapy (dabigatran, apixaban, rivaroxaban, or edoxaban) without any other accompanying anticoagulation for at least one year prior to COVID-19 diagnosis and were still receiving this therapy at the time of diagnosis with another 13,003 patients with COVID-19 who did not take any anticoagulant therapy (either oral or parenteral). Surprisingly, the authors found that prolonged use of NOAC therapy up to the time of COVID-19 diagnosis was not related to better clinical outcomes or reduced hospitalization/rehospitalization rates than those in patients with COVID-19 who were not treated with oral anticoagulants.^42^

### Study limitations

The present meta-analysis has some limitations that must be addressed. The qualities of the included studies had low levels of evidence. Most of them were case reports and case series which may not be representative of all cases. Some of the collected data were incomplete, posing a high risk of prejudice in information. The study comprised a limited number of studies, and each study had its own patient selection bias.

In conclusion, this meta-summary of cases of VTE associated with COVID-19, we identified 10 cases with DVT diagnosed using duplex ultrasonography, compression ultrasonography, or CT venography, and 38 cases with PE based on CTPA with high D-dimer levels and evidence of strain on echocardiography. Patients with VTE often developed common COVID-19 symptoms, including fever, cough, chest pain, fatigue, and shortness of breath. In general, both acute and chronic presentations of VTE are common; however, in individuals with COVID-19, acute VTE can cause serious illness and death.

A high index of suspicion among COVID-19 patients is essential, irrespective of whether they are treated in the general ward or in ICU settings or with anticoagulant therapy prior to or during COVID-19 diagnosis. Our review provides a glimpse into the current management practices used in individual cases of VTE practices and warrants future investigations in larger number of cases.
